# Posttreatment with Ospemifene Attenuates Hypoxia- and Ischemia-Induced Apoptosis in Primary Neuronal Cells via Selective Modulation of Estrogen Receptors

**DOI:** 10.1007/s12640-023-00644-5

**Published:** 2023-05-02

**Authors:** Bernadeta A. Pietrzak, Agnieszka Wnuk, Karolina Przepiórska, Andrzej Łach, Małgorzata Kajta

**Affiliations:** Laboratory of Neuropharmacology and Epigenetics, Department of Pharmacology, Maj Institute of Pharmacology, Polish Academy of Sciences, Smetna Street 12, Krakow, 31-343 Poland

**Keywords:** Ospemifene, SERMs, Neuroprotection, Hypoxia, Ischemia, Primary neurons

## Abstract

Stroke and perinatal asphyxia have detrimental effects on neuronal cells, causing millions of deaths worldwide each year. Since currently available therapies are insufficient, there is an urgent need for novel neuroprotective strategies to address the effects of cerebrovascular accidents. One such recent approach is based on the neuroprotective properties of estrogen receptors (ERs). However, activation of ERs by estrogens may contribute to the development of endometriosis or hormone-dependent cancers. Therefore, in this study, we utilized ospemifene, a novel selective estrogen receptor modulator (SERM) already used in dyspareunia treatment. Here, we demonstrated that posttreatment with ospemifene in primary neocortical cell cultures subjected to 18 h of hypoxia and/or ischemia followed by 6 h of reoxygenation has robust neuroprotective potential. Ospemifene partially reverses hypoxia- and ischemia-induced changes in LDH release, the degree of neurodegeneration, and metabolic activity. The mechanism of the neuroprotective actions of ospemifene involves the inhibition of apoptosis since the compound decreases caspase-3 overactivity during hypoxia and enhances mitochondrial membrane potential during ischemia. Moreover, in both models, ospemifene decreased the levels of the proapoptotic proteins BAX, FAS, FASL, and GSK3β while increasing the level of the antiapoptotic protein BCL2. Silencing of specific ERs showed that the neuroprotective actions of ospemifene are mediated mainly via ESR1 (during hypoxia and ischemia) and GPER1 (during hypoxia), which is supported by ospemifene-evoked increases in ESR1 protein levels in hypoxic and ischemic neurons. The results identify ospemifene as a promising neuroprotectant, which in the future may be used to treat injuries due to brain hypoxia/ischemia.

**Supplementary Information:**

The online version contains supplementary material available at 10.1007/s12640-023-00644-5.

## Introduction

Among brain injuries caused by deficits in oxygen and/or glucose availability, perinatal asphyxia, and stroke can be distinguished. Perinatal asphyxia is a serious condition that affects up to 2% of all newborns born alive and may lead to death in 15–20% of them. Furthermore, prolonged hypoxia may lead to myocardiocyte dysfunction, in turn causing cardiac failure, which results in brain and peripheral organ ischemia. It is estimated that one-fourth of babies that survive these episodes suffer from hypoxic-ischemic encephalopathy (Gebregziabher et al. 2020). Currently, the most commonly used therapy is hypothermia, which has many side effects (Locci et al. 2020). For adults, over 13.7 million people worldwide suffer from stroke yearly, and for 5.5 million of them, the incident leads to death (Johnson et al. 2019). Ischemic stroke (the stroke resulting from a blood vessel clog that disables blood supply) constitutes the majority of strokes (~ 71%) (Feigin et al. 2018). Currently, the main approach to treating ischemic stroke is unblocking the clogged vessel. This treatment can be accomplished in two distinct ways: pharmacologically (utilizing recombinant tissue plasminogen activator (rtPA)) and mechanically (with surgical thrombectomy). Nonetheless, the abovementioned therapies have serious limitations and side effects; e.g., rtPA must be used at a maximum of 4.5 h from the beginning of the episode, and its usage often leads to cerebral hemorrhages, which increase the risk of death (Vivien et al. 2011). Concerning mechanical thrombectomy, the freed clog may block other arteries, such as those in the heart or lungs (Fukuta et al. 2017). Moreover, despite the restoration of blood flow, secondary cerebral ischemia/reperfusion injury may often be induced (Shichita et al. 2012).

Thus, there is an urgent need for new neuroprotective strategies for the treatment of cerebrovascular accidents. One such recent approach is based on the neuroprotective properties of estrogen receptors (ERs). Natural ligands of ERs are steroid hormones belonging to a group of estrogens. Although estrogens act as effective neuroprotectants, their excessive administration may contribute to the development of endometriosis or hormone-dependent cancers, such as ovarian or breast cancer (Patel et al. 2018a, b). Selective estrogen receptor modulators (SERMs) appear to be a much safer option. SERMs are chemical compounds that act as an ER agonist or antagonist, depending on the tissue. Hence, utilizing SERMs enables the simultaneous acquisition of estrogenic effects in desired organs and protection of other organs from estrogen excess. Previous studies have already shown that both natural SERMs (genistein, daidzein) and synthetic SERMs (raloxifene, tamoxifen, bazedoxifene, and pathway preferential estrogen-1 (PaPE-1)) can protect neurons in and/or in vivo models of cerebrovascular accidents (Kimelberg et al. 2000; Kajta et al. 2007; Wakade et al. 2008; Castelló-Ruiz et al. 2011; Kajta et al. 2013; Rzemieniec et al. 2015; Jover-Mengual et al. 2017; Khanna et al. 2017; Rzemieniec et al. 2018; Selvaraj et al. 2018; Burguete et al. 2019; Wnuk et al. 2021a). The compound tested in the current study is ospemifene, which has been approved for the treatment of dyspareunia and used in the USA since 2013 and in Europe since 2015 (Rutanen et al. 2003; Cagnacci et al. 2020). Ospemifene treatment is well tolerated and presents a good safety profile (Di Donato et al. 2019). Aside from dyspareunia, ospemifene has also been tested in the context of breast cancer therapy (Lubián López 2022). The ability of ospemifene to cross the blood–brain barrier (BBB) has not been studied so far. However, the admetSAR, a free tool for evaluating ADMET (absorption, distribution, metabolism, excretion, and toxicity properties of chemicals), predicted that ospemifene has ability to cross the blood–brain barrier with the probability of 0.7095 (DrugBank database). In addition, during stroke, the BBB is damaged (Bernardo-Castro et al. 2020). This pathology makes it possible for the substances to pass through the blood–brain barrier and to exhibit their intended neuroprotection during brain pathology.

Since ospemifene is successfully used in the pharmacotherapy of urogenital diseases, the current study was aimed at assessing for the first time the neuroprotective capacity of ospemifene in in vitro hypoxia and ischemia models.

## Materials and Methods

### Materials

Phosphate-buffered saline (PBS) was purchased from Biomed Lublin (Lublin, Poland). B27 and neurobasal media were obtained from Gibco (Grand Island, NY, USA). The LDH cytotoxicity detection kit was purchased from TaKaRa (Kusatsu, Japan). Enzyme-linked immunosorbent assay (ELISA) kits for BAX, BCL2, FAS, FASL, GSK3β, ESR1, ESR2, and GPER1 were purchased from Bioassay Technology Laboratory (Shanghai, China). Culture plates were obtained from TPP Techno Plastic Products AG (Trasadingen, Switzerland).* L*-glutamine, fetal bovine serum (FBS), dimethyl sulfoxide (DMSO), 4-(2-hydroxyethyl)-1-piperazineethanesulfonic acid (HEPES), 3-[(3-cholamidopropyl)dimethylammonio]-1-propanesulfonate hydrate (CHAPS), ammonium persulfate, N,N,N′,N′-tetramethylethane-1,2-diamine (TEMED), 2-amino-2-(hydroxymethyl)-1,3-propanediol (TRIZMA base), DL-dithiothreitol, sodium deoxycholate, the protease inhibitor cocktail (ethylenediaminetetraacetic acid-free), radioimmunoprecipitation assay buffer (RIPA) buffer, ospemifene, ormeloxifene, thiazolyl blue tetrazolium bromide, protease inhibitor cocktail for mammalian tissues, and polyornithine were obtained from Sigma-Aldrich (St. Louis, MO, USA). JC-10 was purchased from Abcam (Cambridge, UK). The RNeasy Mini Kit was obtained from Qiagen (Hilden, Germany). A High-Capacity cDNA-Reverse Transcription Kit, TaqMan Gene Expression Master Mix, and TaqMan probes for specific genes encoding *Bax*, *Bcl2*, *Fas*, *Fasl*, *Gsk3b*, *Esr1*, *Esr2*, *Gper1*, *Cyp19a1*, and *Actb* were obtained from Thermo Fisher Scientific (Waltham, MA, USA). Fluoro-Jade C was purchased from Biosensis (Australia). Calcein AM and Hoechst 33342 were purchased from Molecular Probes (Eugene, OR, USA).

### Methods

#### Primary Neocortical Cell Cultures

Primary neocortical cell cultures were established from mouse embryos (CD-1^®^ IGS Swiss mouse, Charles River, Germany) on the 15^th^ day of gestation as previously described (Kajta et al. 2006, 2009a, 2020). Briefly, minced cortices were incubated with 0.1% trypsin for 15 min at 37 °C. The trypsinization process was terminated using fetal bovine serum (FBS). The neuronal cells were centrifuged at 1000 RPM for 5 min and then submerged in medium containing 10% FBS. Then, the cells were seeded on poly-L-ornithine-coated (0.1 mg/ml) plates at a density of 2.0 × 10^5^ cells per cm^2^ in multiwell plates. Cells were cultivated in phenol red-free neurobasal medium containing FBS, B27, *L*-glutamine, and penicillin‒streptomycin antibiotics for 2 days. For the following days, a medium deprived of FBS was utilized. The neuronal cell cultures were kept in a humidified incubator at 37 °C with 5% CO_2_ for 7 days in vitro.

All experiments were conducted according to the principles of the Three Rs in compliance with the European Union Legislation (Directive 2010/63/EU, amended by Regulation (EU) 2019.1010) and the ethical standards in the Guide for the Care and Use of Laboratory Animals (NIH).

#### Experimental Models of Normoxia, Hypoxia, and Ischemia

In this study, models of 18 h of normoxia, hypoxia, or ischemia followed by 6 h of reoxygenation were used. All experiments were started on the 7^th^ day of in vitro culture.

##### Normoxia Model

In the normoxic model, on the day of the experiment, the medium was changed to standard medium, and the cells were incubated for 18 h. After that, the spent medium was replaced with fresh medium for the next 6 h.

##### Hypoxia Model

For the induction of hypoxia, the cell medium was replaced with standard medium, and the cells were placed in a prewarmed and humidified hypoxia modulator incubator chamber with 95% N_2_ and 5% CO_2_ for 18 h. The episode was followed by medium replacement with standard medium. Cells were incubated with fresh medium for 6 h.

##### Ischemia Model

For modeling of ischemic conditions, in contrast to hypoxic conditions, glucose-deprived neurobasal medium was utilized. Cells subjected to ischemia were also placed in the abovementioned hypoxia chamber for 18 h. After the episode, the cell medium was immediately changed to a fully supplemented medium for 6 h.

#### Treatment

**Scheme 1:**
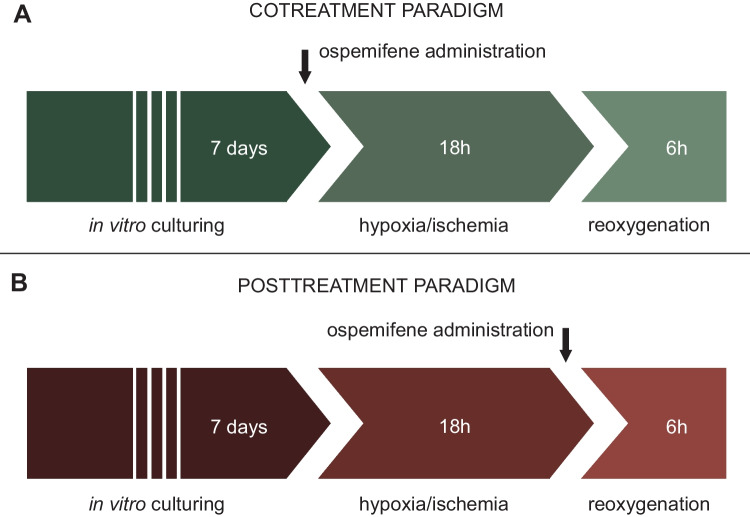
Graphical representation of cotreatment and posttreatment paradigms with ospemifene administration as an example

Ospemifene (0.1–10 μM) was administered in two paradigms: cotreatment and posttreatment (Scheme 1). Ormeloxifene (1–10 μM) was administered in the posttreatment paradigm.

##### Cotreatment

In cotreatment, the tested compound or vehicle was administered at the beginning of the 18 h-long episode of hypoxic/ischemic damage (Scheme 1A). Then, the medium was replaced with fresh medium without the compound/vehicle.

##### Posttreatment

In the posttreatment paradigm, the experiment began after 18 h of hypoxia-/ischemia-induced damage. The tested compound or vehicle was administered for 6 h of reoxygenation (Scheme 1B).

Since posttreatment is much more clinically relevant than cotreatment, it was chosen for the majority of experiments.

#### Measurement of Lactate Dehydrogenase (LDH) Release

LDH release is a hallmark of hypoxia-/ischemia-induced cytotoxicity. For the measurement of the parameters, a cytotoxicity detection kit (TaKaRa, Kusatsu, Japan) was utilized according to the manufacturer’s protocol as previously described (Kajta et al. 2001). After the experiment from each well, cell-free supernatant was collected and then incubated with a proper reagent for 30 min at room temperature. As a result of the reaction, an appropriate amount of formazan salt was produced. The intensity of the red color was measured using the Infinite M200pro microplate reader (Tecan, Mannedorf, Switzerland) at 490 nm and analyzed in i-control software. The absorbance of blanks (no-enzyme controls) was subtracted from each value, and the obtained data were normalized to those of vehicle-treated cells. The obtained results are presented as a percent of the control ± SEM.

#### Apoptosis Evaluation with Measurement of Caspase-3 Activity

Caspase-3 activity is one of the main indicators of apoptotic cell death. For the analysis of caspase-3 activity, cells were first lysed with lysis buffer containing DTT (DL-dithiothreitol, Sigma-Aldrich, St. Louis, MO, USA) and then incubated at 37 °C with the colorimetric substrate Ac-DEVD-*p*NA (Sigma-Aldrich, St. Louis, MO, USA) as previously described (Rzemieniec et al. 2016; Kajta et al. 2019). The substrate was cleaved by caspase-3 to *p*-nitroaniline, and the levels were measured after 60 min using an Infinite M200PRO microplate reader (Tecan, Switzerland) and Tecan i-control software. The blank sample was subtracted from each value, and the obtained data were standardized to the absorbance of the control (DMSO-treated) cells. The results are presented as a percentage of the control ± SEM.

#### Neurodegenerative Cell Staining Utilizing Fluoro-Jade C

Fluoro-Jade C staining was used to label degenerating neurons. The procedure was performed as described by Gu et al. (2014). The culture medium was replaced with reagent (Biosensis, Australia) dissolved in distilled water. Then, the cells were incubated with the mixture for 1 h. The fluorescence intensity was measured at 490 nm excitation and 525 nm emission wavelengths using an Infinite M200PRO microplate reader (Tecan, Switzerland) and Tecan i-control software. The obtained results were normalized to the control cells and are presented as a percent of the control ± SEM. The blank sample was subtracted from each value.

#### Mitochondrial Membrane Potential Assessment Using JC-10

Measurement of mitochondrial membrane potential was performed using a JC-10 Assay Kit (Abcam, Cambridge, UK) as previously described (Przepiórska et al. 2023). JC-10 transpires into mitochondria and polymerizes there in cells with proper mitochondrial membrane potential, forming aggregates with characteristic red fluorescence. Low mitochondrial membrane potential results in a lack of JC-10 transport into mitochondria, leading to JC-10 green fluorescent monomer deposition in the cytoplasm. The cell culture medium was replaced with phosphate-buffered saline (PBS) (Biomed, Poland). After a 15-min incubation, the JC-10 dye solution was added, and the cells were incubated with the reagent for another 30 min. Then, Assay Buffer B was added, and immediate measurements at Ex/Em = 490/525 nm (green fluorescence) and 540/590 (red fluorescence) were performed using the Infinite M200PRO microplate reader (Tecan, Switzerland) and Tecan i-control software. The next step of the analysis was a calculation of the red to green fluorescence ratio. The obtained values were normalized to those of vehicle-treated cells and are expressed as a percent of the control ± SEM.

#### Determination of Cell Metabolic Activity with MTT Staining

MTT staining is based on the oxidoreductase ability to reduce yellow tetrazolium salt to purple formazan salt. The amount of formazan crystals is proportional to cell metabolic activity. Neuronal metabolic activity was determined using thiazolyl blue tetrazolium bromide (Sigma-Aldrich, St. Louis, MO, USA) as previously described (Wnuk et al. 2020, 2021a, 2021b). Briefly, methylthiazolyldiphenyl-tetrazolium bromide (MTT) solution was added to the culture medium for 1 h, and then, to dissolve formazan crystals, the medium was replaced with DMSO. The absorbance was measured at 570 nm in an Infinite M200PRO microplate reader (Tecan, Switzerland) and analyzed with Tecan i-control software. The value of the blank sample was subtracted from each value, and the obtained data were normalized to the absorbance of the control (DMSO-treated) cells. The results are presented as a percentage of the control ± SEM.

#### Staining with Calcein AM and Hoechst 33342

Cells were stained with calcein AM and Hoechst 33342 after 18 h of hypoxia/ischemia and 6 h of reoxygenation, as previously described (Kajta et al. 2009b). Calcein AM, a dye based on intracellular esterase activity, was used to visualize cell viability. Apoptotic cells containing bright blue fragmented nuclei, indicating condensed chromatin, were identified as apoptotic cells using Hoechst 33342. Cells cultured on glass coverslips were washed with PBS and incubated with Hoechst 33342 (0.6 mg/ml) at room temperature (RT) for 5 min. Cells were then washed with PBS and incubated in 2 μM calcein AM in PBS at RT for 10 min. Fluorescence intensity was monitored using a reversed fluorescence microscope (Leica DMIL LED, Leica Camera AG, Wetzlar, Germany) equipped with a Leica Flexacam C3 camera. The mean fluorescence intensity of calcein AM was quantified from 5 to 6 images per group using ImageJ software. The results are presented as a percentage of the control ± SEM.

#### Silencing of *Esr1*, *Esr2*, and *Gper1* Using Small Interfering RNA (siRNA)

*Esr1*, *Esr2*, and *Gper1* gene silencing was performed using specific siRNAs (Santa Cruz Biotechnology, Santa Cruz, CA, USA). Each siRNA was applied separately for 7 h at 50 nM in antibiotic-free medium containing the siRNA transfecting reagent INTERFERin™ as previously described (Kajta et al. 2014, 2020). After transfection, the cell culture medium was changed, and the cells were incubated for 21 h prior to the start of the hypoxic/ischemic experiment. As a control, negative (scrambled) siRNA containing sequences that do not lead to degradation of any known mRNA was used. The effectiveness of mRNA silencing was previously verified through the measurement of specific mRNAs using qPCR (Rzemieniec et al. 2015; Wnuk et al. 2018).

#### RT qPCR Analysis

An RNeasy Mini Kit (Qiagen, Hilden, Germany) and the spin column method were used to extract total RNA from neocortical cell cultures. The quantity of RNA was measured using an ND-1000 UV Vis NanoDrop (Thermo Fisher NanoDrop, Waltham, MA, USA) at 260 nm and 260/280 nm. Immediately after isolation, the extracted RNA was reverse-transcribed with a High-Capacity cDNA Reverse Transcription Kit (Thermo Fisher Scientific, Waltham, MA, USA) with the T100 Thermal Cycler (Bio-Rad, Hercules, CA, USA). The obtained cDNA was then stored at − 20 °C. The cDNA was amplified by quantitative polymerase chain reaction (qPCR) using FastStart Universal Probe Master (Roche, Switzerland) that included TaqMan Gene Expression Assays (Thermo Fisher Scientific, Waltham, MA, USA) specific for *Bax*, *Bcl2*, *Fas*, *Fasl*, *Gsk3b*, *Esr1*, *Esr2*, *Gper1*, *Cyp19a1*, *Hprt*, *Gapdh*, and *Actb*. The amplification mixture in a total volume of 20 μl contained the following: 10 μl of FastStart Universal Probe Master, 8 μl of RNase free water, 1 μl of cDNA template, and 1 μl of the TaqMan gene expression Assay Mix. The procedure was performed using the following steps: 2 min at 50 °C, 10 min at 95 °C, and 40 cycles of 15 s at 95 °C and 1 min at 60 °C, with the CSX Real-Time system (Bio-Rad, Hercules, CA, USA) as previously described (Wnuk et al. 2016, 2018, 2019). Then, the delta Ct method was utilized to analyze the obtained data. To choose the reference gene, we used the following algorithms: geNorm, NormFinder, BestKeeper, and delta Ct, which identified *Actb* as the most stable reference gene among all experimental groups.

#### Enzyme-Linked Immunosorbent Assays (ELISAs)

To determine the protein expression of BAX, BCL2, FAS, FASL, GSK3β, ESR1, ESR2, and GPER1, we used enzyme-linked immunosorbent mouse-specific assays (ELISAs; Bioassay Technology Laboratory, China) according to the manufacturer’s instructions, as previously described (Kajta et al. 2017; Rzemieniec et al. 2019). Values obtained by the absorbance measurement at 450 nm were correlated with the amount of specific protein. For the measurement of the total protein concentration, Bradford reagent (Bio-Rad Protein Assay, Hercules, CA, USA) and bovine serum albumin (as a standard) were utilized. The results are presented as a percentage of the control ± SEM.

#### Data Analysis

The statistical tests were performed on raw data and are expressed as the mean absorbance or fluorescence units per well containing 50,000 cells for the LDH, caspase-3, Fluoro-Jade C, JC-10, and MTT assays; the fluorescence units per 1.5 million cells for qPCR; and the pg of BAX, BCL2, FAS, FASL, GSK3β, ESR1, ESR2, and GPER1 per µg of total protein for the ELISAs. Leven’s test for homogeneity of variances, followed by one-way analysis of variance (ANOVA), was used to determine the overall significance. The differences between the control and experimental groups were assessed with a post hoc Newman‒Keuls test. Significant differences were symbolized as follows: ^*^p < 0.05, ^**^p < 0.01, and ^***^p < 0.001 (compared to the control groups), ^#^*p* < 0.05, ^##^*p* < 0.01, and ^###^*p* < 0.001 (compared to the cells subjected to hypoxia), and ^^^*p* < 0.05, ^^^^*p* < 0.01, and ^^^^^*p* < 0.001 (compared to the cultures exposed to ischemia). The results are expressed as the mean ± SEM of the number of experiments and replicates indicated below the corresponding figures.

## Results

### Ospemifene Decreased Hypoxia- and Ischemia-Induced LDH Release in the Posttreatment Paradigm, but Ormeloxifene Stimulated LDH Release in Hypoxic and Ischemic Neurons

Measurement of LDH release was performed to assess the extent of hypoxia/ischemia-induced cytotoxicity. Eighteen hours of hypoxia or ischemia followed by 6 h of reoxygenation resulted in an increase in LDH release to 312-315% or 280% of the normoxic value, respectively.

**Fig. 1:**
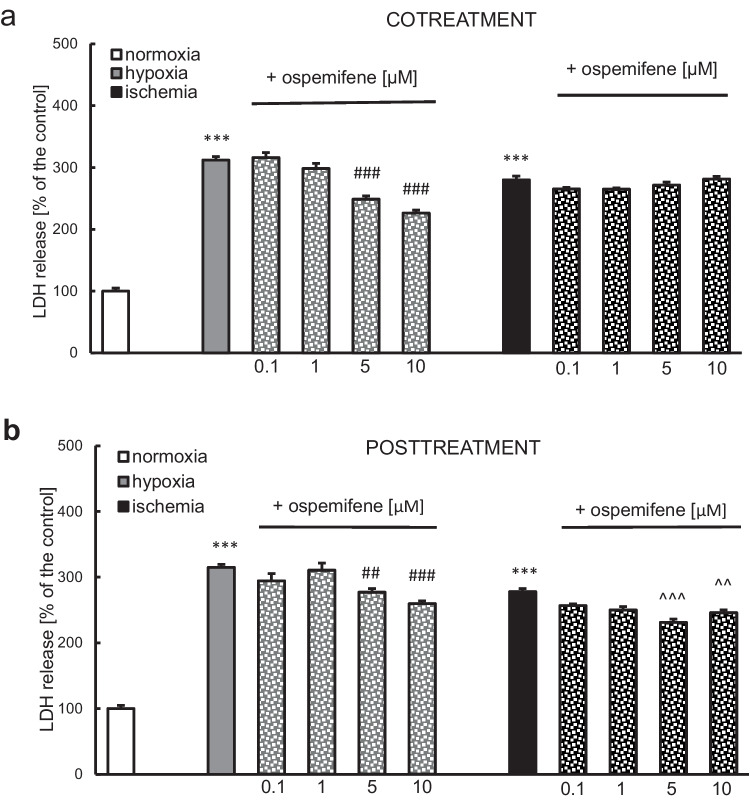
Ospemifene reduced hypoxia- and ischemia-induced LDH release in the posttreatment paradigm. Ospemifene (0.1, 1, 5, and 10 μM) was applied in the cotreatment paradigm (**a**) and posttreatment paradigm (**b**). The results are presented as a percentage of the normoxic control. Each bar represents the mean ± SEM of three independent experiments. The number of replicates ranged from 20 to 30. ^***^p < 0.001 compared to the normoxic cultures; ^##^*p* < 0.01 and ^###^*p* < 0.001 compared to the cultures exposed to hypoxia; ^^^^*p* < 0.01 and ^^^^^*p* < 0.001 compared to the cultures exposed to ischemia

In the hypoxic model, ospemifene at concentrations of 5 and 10 μM effectively decreased LDH release from 312 to 248% and 226% of the normoxic control in cotreatment (Fig. 1a) and from 315 to 277% and 260% of the normoxic control in the posttreatment paradigm (Fig. 1b). Furthermore, ospemifene (5 and 10 μM) administration after the episode effectively diminished ischemia-induced LDH release, resulting in a change in this parameter from 278% of the control to 231% and 246%, respectively (Fig. 1b).

In these models, ospemifene concentrations of 0.1 and 1 μM were ineffective. Concentrations of 5 and 10 μM did not have any protective effects in the ischemic model when the compound was administered in a cotreatment paradigm.

Ospemifene at concentrations of 0.1, 1, 5, and 10 μM, either in the co- or posttreatment paradigm, did not change LDH release under normoxic conditions (Supplementary Materials, Tables S1 and S2).

Ormeloxifene (1, 5, and 10 μM) was applied in the posttreatment paradigm. Posttreatment with ormeloxifene (10 μM) further increased both hypoxia- and ischemia-induced LDH release, while concentrations of 1 and 5 μM did not alter this parameter in either model (Supplementary Materials, Fig. S1). In normoxic conditions, posttreatment with ormeloxifene did not affect LDH release (Supplementary Materials, Table S3).

### Ospemifene Decreased Elevated Caspase-3 Activity in the Hypoxic Model in Both Cotreatment and Posttreatment Paradigms, but Ormeloxifene Stimulated Caspase-3 Activity in Hypoxic Neurons

Caspase-3 activity is a well-known indicator of apoptotic cell death. Hypoxia and ischemia increase caspase-3 activity to approximately 170% and 145% of the normoxic value, respectively.

**Fig. 2:**
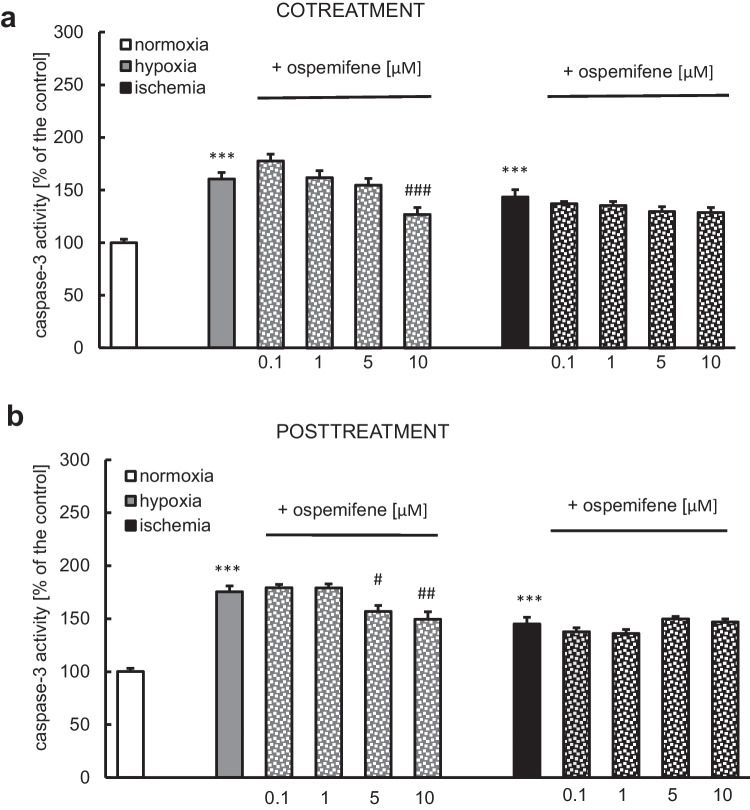
Ospemifene decreased elevated caspase-3 activity in the hypoxic model in both co-treatment and posttreatment paradigms. Ospemifene (0.1, 1, 5, and 10 μM) was applied in the cotreatment paradigm (**a**) and posttreatment paradigm (**b**). The results are presented as a percentage of the normoxic control. Each bar represents the mean ± SEM of three independent experiments. The number of replicates ranged from 20 to 30. ^***^p < 0.001 compared to the normoxic cultures; ^#^*p* < 0.05, ^##^*p *< 0.01 and ^###^*p* < 0.001 compared to the cultures exposed to hypoxia

Application of ospemifene at a concentration of 10 μM decreased caspase-3 activity in the hypoxic model in either paradigm (reduction from 160 to 127% of the normoxic value in the cotreatment paradigm and from 175 to 150% in the posttreatment paradigm) (Fig. 2a, b). The concentration of 5 μM was effective only in the posttreatment paradigm and caused caspase-3 activity to decrease from 175 to 157% of the normoxic control (Fig. 2b). In the ischemic model, ospemifene showed no effects in either of the utilized paradigms (Fig. 2a, b).

Ospemifene (0.1, 1, 5, and 10 μM), administered either in the co- or posttreatment paradigm, did not change caspase-3 activity under normoxic conditions (Supplementary Materials, Tables S1 and S2).

Since the posttreatment paradigm reflects clinical aspects much better than cotreatment and ospemifene is even more protective in this paradigm, we decided to use the posttreatment model for the following experiments. The most effective concentrations, i.e., 5 and 10 μM, were chosen for the next experiments.

Posttreatment with ormeloxifene (1, 5, and 10 μM) further increased hypoxia-induced caspase-3 activity and did not alter ischemia-induced caspase-3 activity (Supplementary Materials, Fig. S2). In normoxia, ormeloxifene had no effect on caspase-3 activity (Supplementary Materials, Table S3).

### Ospemifene Reduced the Neuronal Cell Degeneration Induced by Hypoxia and Ischemia

**Fig. 3:**
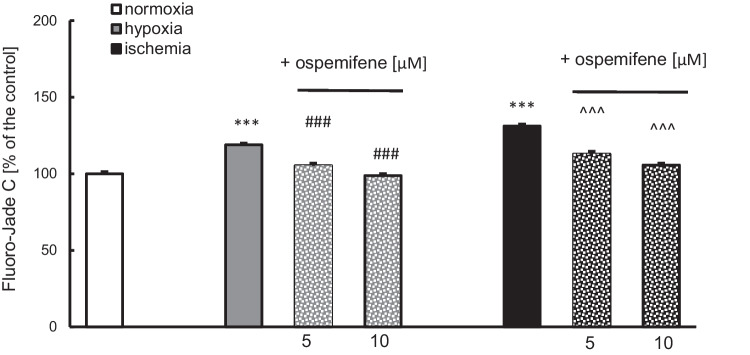
Posttreatment with ospemifene (5 and 10 μM) diminished neuronal cell degeneration (in terms of Fluoro-Jade C staining) induced by hypoxia and ischemia. The results are presented as a percentage of the normoxic control. Each bar represents a mean ± SEM. The number of replicates ranged from 12 to 24. ^***^p < 0.001 compared to the normoxic cultures; ^###^*p* < 0.001 compared to the cultures exposed to hypoxia; ^^^^^*p* < 0.001 compared to the cultures exposed to ischemia

Fluoro-Jade C labeling was used to assess the degree of neurodegeneration. Ospemifene (5 and 10 μM) effectively reduced hypoxia-induced cell degeneration from 118% of the normoxic control to 105% (5 μM) and 99% (10 μM) (Fig. 3). Moreover, the tested compound normalized the ischemia-induced increase in neurodegeneration, from 131% of the normoxic control to 113% in the case of 5 μM ospemifene and 106% in the case of 10 μM ospemifene (Fig. 3).

Ospemifene administration showed no effects on the degree of neurodegeneration under normoxic conditions (Supplementary Materials, Table S2).

### Ospemifene Partially Restored the Lost Mitochondrial Membrane Potential in the Ischemic Model

**Fig. 4:**
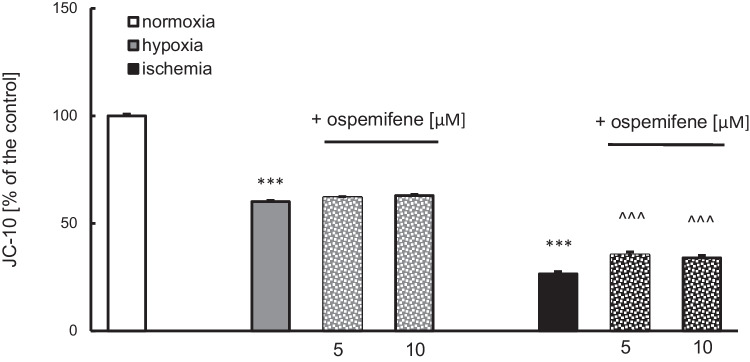
Posttreatment with ospemifene (5 and 10 μM) partially restored the lost mitochondrial membrane potential in the ischemic model. The results are presented as a percentage of the normoxic control. Each bar represents the mean ± SEM of three independent experiments. The number of replicates ranged from 15 to 20. ^***^p < 0.001 compared to the normoxic cultures; ^^^^^*p* < 0.001 compared to the cultures exposed to ischemia

JC-10 dye was used to measure mitochondrial membrane potential. Both hypoxia and ischemia induced a loss of mitochondrial membrane potential to 60% and 24% of the control value, respectively (Fig. 4). Ospemifene at concentrations of 5 and 10 μM partially restored the lost potential in the ischemic model (35% and 33% of the normoxic value, respectively) but showed no effects under hypoxic conditions (Fig. 4).

Ospemifene itself (5 and 10 μM) did not alter this parameter under normoxic conditions (Supplementary Materials, Table S2).

### Ospemifene Restored Cell Metabolic Activity, Which Was Decreased by Hypoxia and Ischemia

**Fig. 5:**
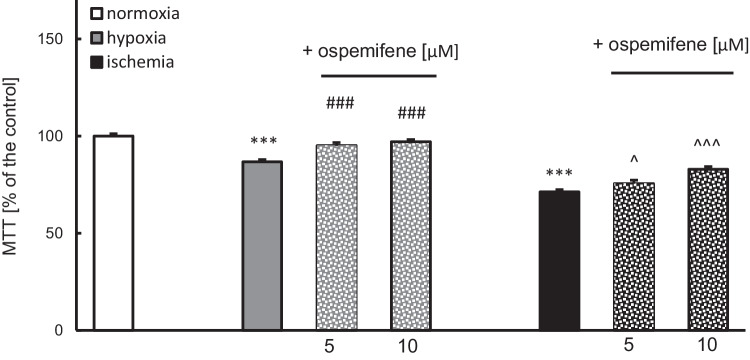
Posttreatment with ospemifene elevates cell metabolic activity that was decreased by hypoxia and ischemia. The results are presented as a percentage of the normoxic control. Each bar represents the mean ± SEM of three independent experiments. The number of replicates ranged from 10 to 20. ^***^p < 0.001 compared to the normoxic cultures; ^###^*p* < 0.001 compared to the cultures exposed to hypoxia; ^^^*p* < 0.05 and ^^^^^*p* < 0.001 compared to the cultures exposed to ischemia

Hypoxia and ischemia diminished cell metabolic activity to 86% and 71% of the normoxic value, respectively (Fig. 5). Ospemifene administration increased the abovementioned parameter value to 95% (5 μM) and 97% (10 μM) in the hypoxia model and to 75% (5 μM) and 83% (10 μM) in the ischemia model (Fig. 5).

Under normoxic conditions, 5 and 10 μM ospemifene did not cause profound changes in cell metabolic activity (Supplementary Materials, Table S2).

For further analyses, 10 μM ospemifene was selected because it caused more pronounced neuroprotection than 5 μM ospemifene.

### Ospemifene Increased Reduced Cell Viability and Lowered Apoptotic Body Formation

**Fig. 6:**
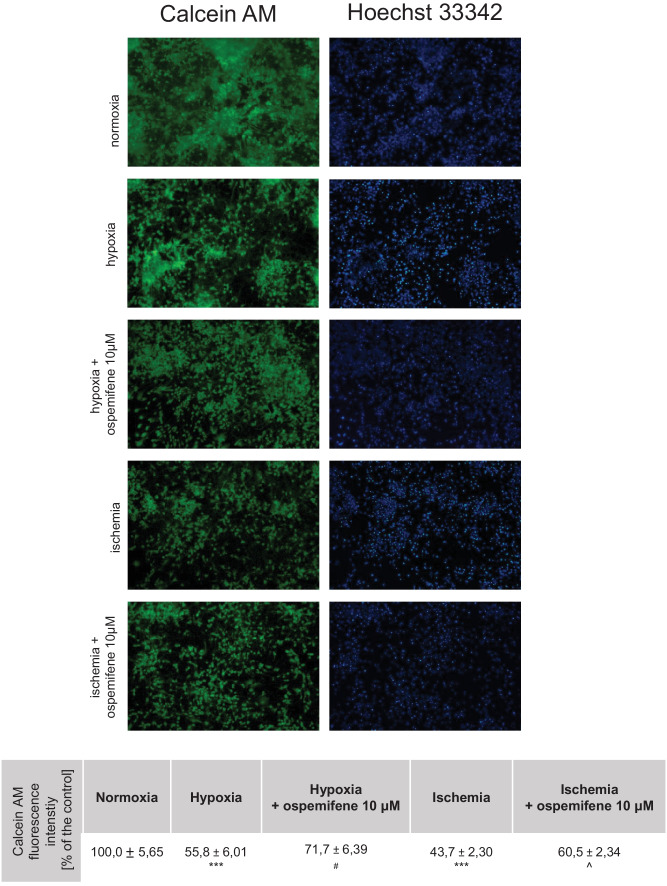
Hypoxia and ischemia decrease cell viability and increase apoptotic body formation. Ospemifene (10 μM) partially reverses these alterations. The results in the table are presented as a percentage of the normoxic control. Each value represents the mean ± SEM of the mean fluorescence intensity from 5 to 6 photos per group. ^***^p < 0.001 compared to the normoxic cultures; ^#^*p* < 0.05 compared to the cultures exposed to hypoxia; ^^^*p* < 0.05 compared to the cultures exposed to ischemia

As indicated by calcein AM staining, hypoxia and ischemia decreased cell viability to 56% and 44% of the normoxic control, respectively (Fig. 6). Ospemifene (10 μM) partially reversed the observed effects in both hypoxia and ischemia (72% and 61% of the normoxic control, respectively) but did not alter the parameter in normoxia (Supplementary Materials, Table S2).

Both hypoxia and ischemia increased apoptotic body formation, visible as bright blue fragmented nuclei, indicating condensed chromatin. Administration of ospemifene (10 μM) reduced the number of apoptotic bodies in hypoxic and ischemic conditions (Fig. 6).

### Ospemifene Altered the mRNA and Protein Levels of Apoptosis-Related Factors

**Fig. 7:**
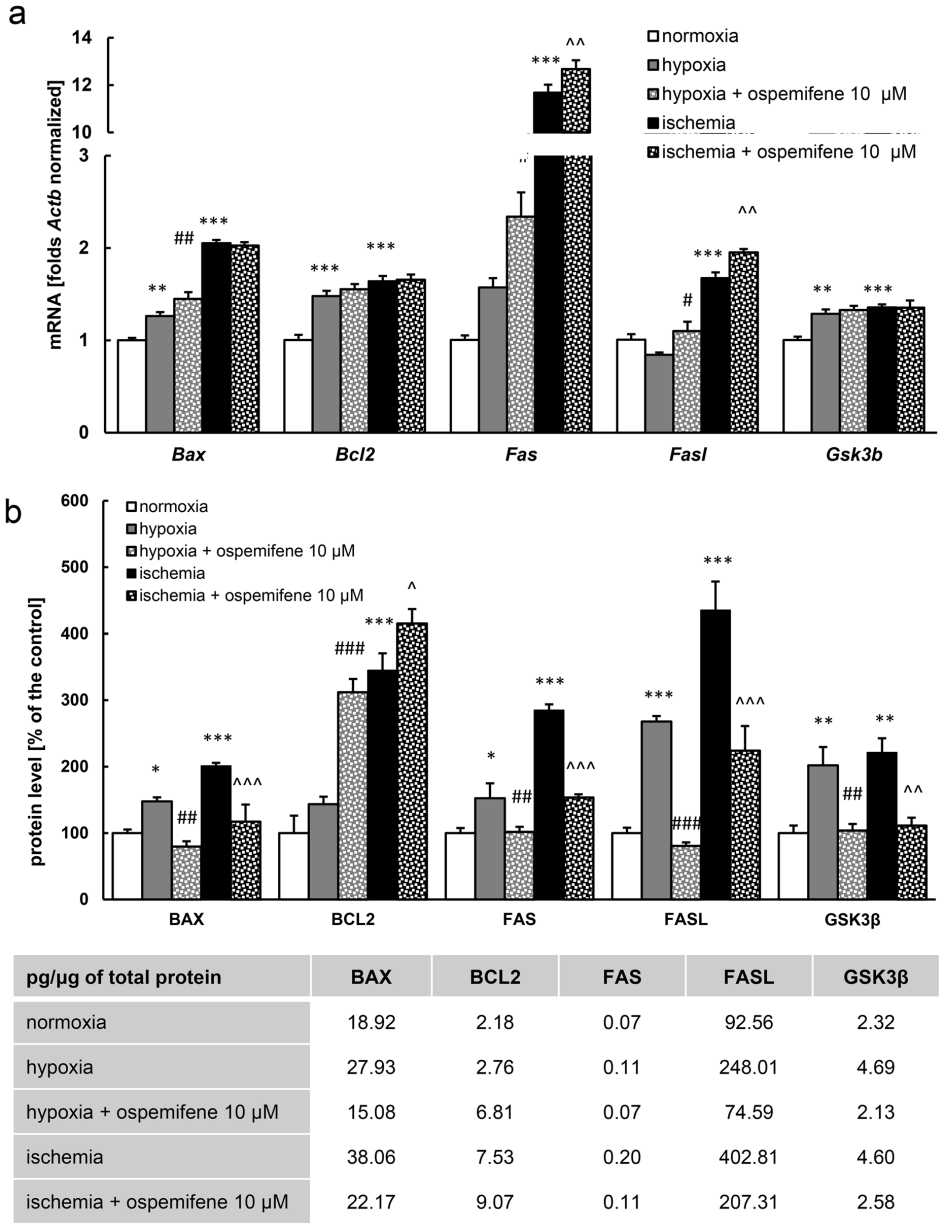
Hypoxia and ischemia as well as posttreatment with ospemifene (10 μM) affected the levels of apoptosis-related mRNAs and protein levels. The results are presented as a percentage of the normoxic control. Each bar represents the mean ± SEM of 4 to 5 replicates per group. ^*^p < 0.05, ^**^p < 0.01, and ^***^p < 0.001 compared to the normoxic cultures; ^#^*p* < 0.05, ^##^*p* < 0.01, and ^###^*p* < 0.001 compared to the cultures exposed to hypoxia; ^^^*p* < 0.05, ^^^^*p* < 0.01, and ^^^^^*p* < 0.001 compared to the cultures exposed to ischemia

Under the experimental conditions, the mRNA levels of antiapoptotic *Bcl2* and proapoptotic *Gsk3b* are altered in similar ways, i.e., both hypoxia and ischemia significantly increased their levels (for *Bcl2*, 1.48-fold increase in hypoxia and 1.64-fold increase in ischemia; for *Gsk3b*, 1.29- and 1.36-fold increase in hypoxia and ischemia, respectively). The application of ospemifene showed no influence on the expression of the abovementioned genes in either the hypoxic or ischemic model (Fig. 7a). Hypoxia also increased the levels of proapoptotic *Bax* (1.26-fold) but did not affect *Fas* and *Fasl* levels, whereas the ischemic model was characterized by an increase in the mRNA levels of *Bax* (2.05-fold), *Fas* (11.68-fold), and *Fasl* (1.67-fold). Ospemifene (10 μM) caused a further increase in *Bax* (from 1.26- to 1.45-fold) and *Fas* (from 1.57- to 2.44-fold) expression in the hypoxic model as well as *Fas* (from 11.68- to 12.68-fold) and *Fasl* (from 1.67- to 1.95-fold) expression in the ischemic model (Fig. 7a).

Eighteen hours of either hypoxia or ischemia followed by 6 h of reoxygenation increased the protein levels of proapoptotic BAX (148% and 201% of the control in hypoxia and ischemia, respectively), FAS (152% and 285% of the normoxic control in hypoxia and ischemia, respectively), FASL (268% and 435% of the normoxic control in hypoxia and ischemia, respectively), and GSK3β (202% and 221% of the normoxic control in hypoxia and ischemia, respectively) (Fig. 7b). Administration of 10 μM ospemifene in both hypoxic and ischemic models normalized the protein levels of all of the abovementioned proapoptotic proteins to levels comparable to the normoxic values (Fig. 7b). Moreover, in hypoxia, ospemifene (10 μM) enhanced the antiapoptotic BCL2 protein level (312% of the normoxic control) (Fig. 7b). The level of BCL2 was also elevated in response to ischemia (345% of the normoxic control) (Fig. 7b). Additionally, a further increase was observed under the influence of 10 μM ospemifene (415% of the normoxic control) (Fig. 7b).

In normoxia, ospemifene administration influenced the expression of neither of the abovementioned apoptosis-related mRNAs nor proteins (Supplementary Materials, Tables S4 and S5).

### Ospemifene Altered the mRNA and Protein Levels of Estrogen Signaling-Related Factors

**Fig. 8:**
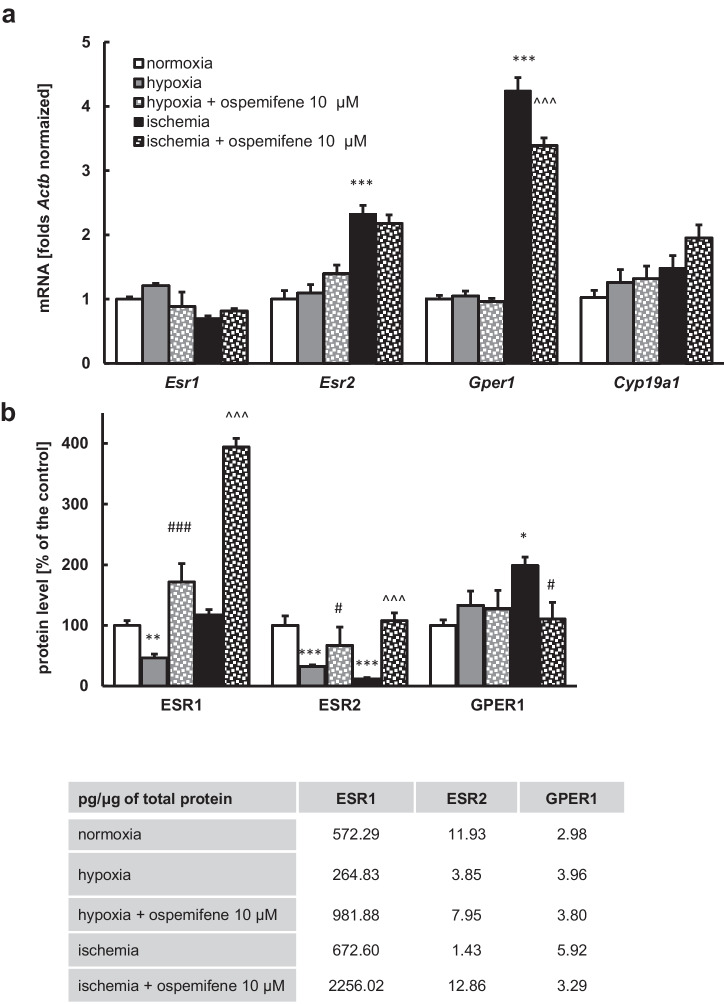
The effects of 18 h of hypoxia or ischemia followed by 6 h of reoxygenation as well as 10 μM ospemifene administration on the mRNA and protein levels of estrogen signaling-related factors. Ospemifene was administered at the beginning of the reoxygenation period, i.e., in the posttreatment paradigm. Each bar represents a mean ± SEM. The number of replicates per group ranged from 4 to 5. ^*^p < 0.05, ^**^p < 0.01, and ^***^p < 0.001 compared to the normoxic cultures; ^#^*p* < 0.05 and ^###^*p* < 0.001 compared to the cultures exposed to hypoxia; ^^^^^*p* < 0.001 compared to the cultures exposed to ischemia

In primary neocortical cell cultures subjected to 18 h of ischemia followed by a 6 h reoxygenation period, we observed a significant increase in *Esr2* and *Gper1* mRNA expression (2.33- and 4.24-fold, respectively) (Fig. 8a). Administration of ospemifene at a concentration of 10 μM diminished, elevated in response to the ischemia, *Gper1* mRNA expression (from 4.24- to 3.91-fold) (Fig. 8a). The *Esr1* and *Cyp19a1* genes did not show altered expression in response to hypoxic/ischemic damage or treatment with ospemifene.

For protein expression, exposure to the hypoxic model led to a decrease in ESR1 (46% of the normoxic control) and ESR2 (32% of the normoxic control) protein levels. Cells that underwent ischemia showed a profound decrease in ESR2 (12% of the normoxic control) protein expression and an elevation of GPER1 (199% of the normoxic control) protein expression (Fig. 8b). Ospemifene (10 μM) administered 18 h after the beginning of the episode (in the reoxygenation period) increased ESR1 levels in both hypoxic and ischemic models (172% and 394% of the normoxic control) (Fig. 8b). Moreover, ospemifene (10 μM) normalized the levels of ESR2 diminished in response to both hypoxia and ischemia as well as normalized the GPER1 level elevated in response to ischemia (Fig. 8b).

Under normoxic conditions, administration of ospemifene did not affect the expression of the abovementioned estrogen signaling-related mRNAs and proteins, except for the ESR2 level (Supplementary Materials, Tables S4 and S5).

### Impact of Specific siRNAs on Ospemifene-Evoked Neuroprotection in Hypoxia–Ischemia-Induced Damage

#### Hypoxic Damage

**Fig. 9:**
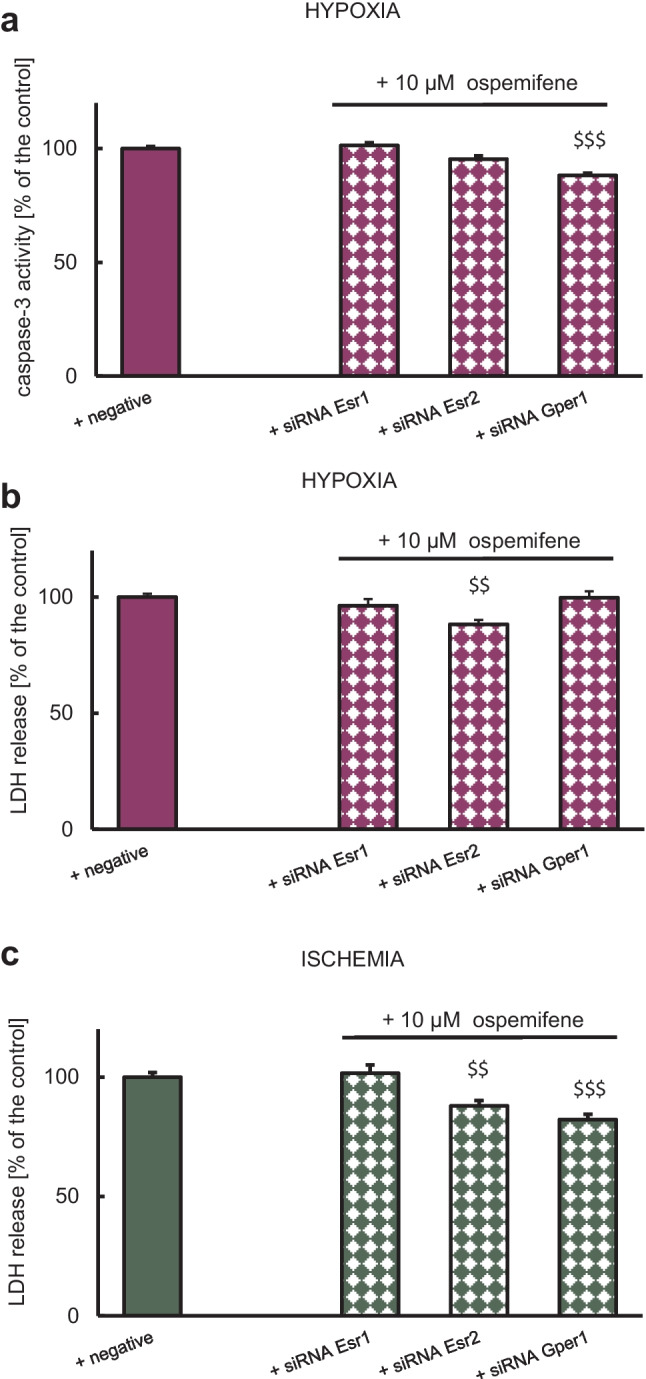
The effects of siRNA-induced silencing of *Esr1*, *Esr2*, or *Gper1* on caspase-3 activity and LDH release in response to hypoxia (**a, b**) and ischemia (**c**) in combination with ospemifene (10 μM) administration. Each bar represents the mean ± SEM of two independent experiments. The number of replicates ranged from 10 to 20. ^$$^*p* < 0.01 and ^$$$^*p* < 0.001 compared to the cultures exposed to negative (scrambled) siRNA and hypoxia or ischemia, respectively

Since ospemifene is neuroprotective in hypoxic neurons in terms of LDH release and caspase-3 activity (Figs. 1 and 2), we tested the involvement of specific ER types in the observed effects using specific siRNAs targeting *Esr1*, *Esr2*, and *Gper1*. In the cells undergoing hypoxia, *Esr1* and *Esr2* siRNAs diminished ospemifene-induced neuroprotection measured by caspase-3 activity, and *Esr1* and *Gper1* siRNAs limited neuroprotective potential of ospemifene measured by LDH release. In the cells subjected to hypoxia and then treated with ospemifene, *Esr1* and *Esr2* siRNAs increased caspase-3 activity to 101% and 95% of the negative hypoxic control, respectively (Fig. 9a). Moreover, in these cells, *Esr1* and *Gper1* siRNAs increased LDH release to 96% and 100% of the negative hypoxic control, respectively (Fig. 9b).

#### Ischemic Damage

As mentioned above, ospemifene is neuroprotective in an ischemic model in terms of LDH release (Fig. 1). In the cells subjected to ischemia, *Esr1* silencing abrogated the ospemifene-induced decrease in LDH release by increasing this parameter to 102% of the negative ischemic control (Fig. 9c). In the ischemic model, ospemifene did not affect caspase-3 activity; therefore, the impact of specific siRNAs has not been assessed.

## Discussion

There is an urgent need for novel therapeutic strategies to treat conditions such as perinatal asphyxia and brain stroke. Since the available therapies are insufficient, researchers have put more effort into solving this pressing issue. According to the obtained data, ospemifene appears to be a promising pharmaceutical tool that can safely be used to treat injuries from brain hypoxia and ischemia. In this study, we demonstrated for the first time that posttreatment with ospemifene in primary neocortical cell cultures subjected to 18 h of hypoxia and/or ischemia followed by 6 h of reoxygenation has robust neuroprotective potential. Ospemifene partially reversed the hypoxia/ischemia-induced increase in LDH release and neurodegeneration, as well as the hypoxia/ischemia-evoked loss of metabolic activity. The mechanism of neuroprotective actions of ospemifene involves the inhibition of apoptosis since the compound lowers caspase-3 overactivity during hypoxia and enhances mitochondrial membrane potential during ischemia. Moreover, in both models of neuronal damage, ospemifene decreased the levels of the proapoptotic proteins BAX, FAS, FASL, and GSK3β while increasing the level of the antiapoptotic protein BCL2. Silencing of specific ERs suggested that the neuroprotective actions of ospemifene are mediated mainly via ESR1 (during hypoxia and ischemia) and GPER1 (during hypoxia), which is supported by ospemifene-evoked increases in ESR1 protein levels in hypoxic and ischemic neurons. Despite emerging evidence on the protective roles of certain SERMs against neurodegeneration, our data indicate that the neuroprotective capacity in such conditions is not attributed to all SERMs, as evidenced by testing ormeloxifene (supplementary data).

Neuronal cells subjected to hypoxia and ischemia die mainly in necrotic and apoptotic manners. Apoptosis is the basic mechanism of neuronal cell death that accompanies stroke and perinatal asphyxia. It is mainly because many apoptosis-related factors are located in mitochondria, and these organelles play a prominent role in cell metabolism that is depleted during oxygen deprivation. Cell membrane permeability is a hallmark of necrotic cell death, and in our study, the quantitative assessment of this process was performed by measuring the amount of LDH secreted outside the cell. We demonstrated that ospemifene protects neurons from hypoxia- and ischemia-induced increases in LDH activity and reduces caspase-3 activity (estimated to assess apoptosis) elevated due to hypoxia. Similar effects have been observed previously with other SERMs (e.g., bazedoxifene, raloxifene, and tamoxifen) or phytoestrogens (genistein, daidzein, and equol-daidzein metabolite), which possess SERM properties (Kajta et al. 2007, 2013; Wakade et al. 2008; Schreihofer and Redmond 2009; Rzemieniec et al. 2018). Nevertheless, the abovementioned substances were used in pre- or cotreatment paradigms, which do not reflect clinical utilization as a posttreatment paradigm does. Importantly, in the current study, ospemifene induced a significant neuroprotective effect when administered to the system several hours after the onset of the hypoxic-ischemic episode, which indicates the clinical potential of this compound. In this study, we also showed that ospemifene protects cells from neurodegeneration, which is similar to the effects previously demonstrated for tamoxifen and raloxifene in models of retinal ganglion cell and photoreceptor degeneration (Wang et al. 2017; Getter et al. 2019; Jiang et al. 2019).

A number of studies have proven that mitochondrial dysfunction plays an important role in the pathophysiology of neurodegenerative diseases, both acute (e.g., stroke) and chronic, e.g., Parkinson’s disease or Alzheimer’s disease (Soane et al. 2007). Improper mitochondrial function leads to several consequences, such as oxidative stress, disturbance of calcium ion homeostasis, induction of apoptosis, and metabolic failure (Soane et al. 2007). In the present study, hypoxia and ischemia decreased both the number of metabolically active cells and mitochondrial membrane potential. These effects were normalized by ospemifene, except for the hypoxia-induced decrease in mitochondrial membrane potential. The decreased mitochondrial membrane potential under hypoxic and ischemic conditions is a well-known phenomenon (Rzemieniec et al. 2015, 2018; Park et al. 2019; Ye et al. 2021). In addition to estradiol (Wang et al. 2001; Guo et al. 2010; Zhao et al. 2016), other SERMs, including raloxifene and bazedoxifene (in the cotreatment paradigm), are able to partially normalize this parameter (Rzemieniec et al. 2018).

The apoptotic pathway consists of several protein cascades, among which proapoptotic molecules such as BAX, BAD, BIM, JNK, GSK3β, and FAS as well as antiapoptotic BCL2 and BCLXL play important roles (Wnuk and Kajta 2017). In the current study, hypoxia and ischemia increased the expression of proapoptotic BAX, FAS, FASL, and GSK3β. Similar effects of hypoxia and ischemia have been observed in our previous studies performed with different time paradigms (6 h of hypoxia/ischemia and 18 h of reoxygenation) (Wnuk et al. 2021a, 2021b). The protein level of antiapoptotic BCL2 did not change in response to hypoxia but was increased in the ischemic model. The molecular mechanism of interaction between BCL2 and BAX relies on BCL2-induced conformational changes of BAX that prevent its oligomerization, which is necessary to form pores in the mitochondrial membrane (Dlugosz et al. 2006); in this way, the decrease in the BCL2/BAX ratio induces apoptosis. Upregulation of BCL2 in the ischemic model may seem surprising, but similar effects have already been observed in other cellular models of brain hypoxia and ischemia as well as Alzheimer’s and Huntington’s diseases (Satou et al. 1995; O’Barr et al. 1996; Kitamura et al. 1998; Sulejczak et al. 2004; Sassone et al. 2013; Wnuk et al. 2021a, 2021b; Przepiórska et al. 2023). The observed phenomenon is likely a survival mechanism. Ospemifene normalized all the mentioned proapoptotic protein levels and increased BCL2 levels in both used models. In general, estrogens are known to inhibit proapoptotic JNK and GSK3β kinase pathways, decrease the expression of proapoptotic proteins, such as BAX, BAD, or BIM, and increase antiapoptotic BCL2 and BCLXL expression (Arevalo et al. 2015; Wnuk and Kajta 2017; Raghava et al. 2017). A similar effect of upregulation of BCL2 and downregulation of BAX has been described previously for daidzein and genistein, which are phytoestrogens with SERM properties (Liu et al. 2017; Li et al. 2017). PaPE-1, a newly synthesized ER ligand that selectively acts on membrane ERs, has also recently been shown to reduce BAX, FAS, FASL, and GSK3β levels and upregulate BCL2 in response to hypoxia and ischemia (Wnuk et al. 2021a). The differences between the observed mRNA and protein levels may result from posttranscriptional degradation of mRNA, which may also contribute to the neuroprotective actions of the tested compound.

Ospemifene has a similar affinity for ESR1 (K_*i*_ = 380 nM) and ESR2 (K_*i*_ = 410 nM), but its binding to these receptors is weaker than that of estradiol (K_*i*_ = 10 and 5 nM, respectively) (Unkila et al. 2013). However, affinity does not necessarily determine the involvement of a given type of receptor in a particular effect observed under the influence of a ligand, as we evidenced using specific siRNAs. According to our results, in the cells subjected to ischemia and transfected with *Esr1* siRNA, but not *Esr2* or *Gper1* siRNAs, ospemifene lost its neuroprotective property in terms of LDH release, which suggests the involvement of ESR1 in ospemifene-evoked neuroprotection against ischemic damage. For the model of hypoxia, in the cells transfected with all siRNAs used, ospemifene lost its neuroprotective capacity in terms of LDH release (*Esr1* and *Gper1* silencing) and caspase-3 activity (*Esr1* and *Esr2* silencing). Therefore, all studied ERs appeared to be involved in the neuroprotective action of ospemifene against hypoxic damage, although the measures of neuroprotection were different. Taking into account the LDH parameter, which reflects the final effect independent of preceding processes, such as caspase-3-dependent apoptosis, one may suggest that ESR1 plays a crucial role in ospemifene-evoked neuroprotection against hypoxic and ischemic damage, whereas GPER1 mediates the neuroprotective action of ospemifene only during hypoxia.

Ospemifene has thus far been tested on the U2OS osteosarcoma line, where similar to our results, the protective effect of ospemifene against etoposide-induced apoptosis was mediated primarily by ESR1 (Kallio et al. 2008). The protective effects of estrogens and SERMs may have different bases that depend on the type of ligand, the level of coregulators (coactivators and corepressors) in the cells and even their location in the body (Patel et al. 2018a, b). Previously, we demonstrated that the SERMs raloxifene and bazedoxifene executed ESR1-mediated neuroprotection against hypoxic damage in cortical and hippocampal neurons (Rzemieniec et al. 2015, 2018). We also showed key roles of ESR2 and GPER1 in the neuroprotective effect of daidzein, a phytoestrogen that possesses SERM properties (Kajta et al. 2013). Moreover, membrane ESR1 and ESR2 were found to mediate the protective effects of PaPE-1 on whole brains (Selvaraj et al. 2018) and hypoxic/ischemic neurons (Wnuk et al. 2021a). 

In our study, a key role of ESR1 in ospemifene neuroprotection is supported by ospemifene-evoked increases in ESR1 protein levels during hypoxia and ischemia. Unfortunately, GPER1 involvement in ospemifene neuroprotection is not reflected by the ospemifene-induced increase in GPER1 protein levels during hypoxia. Intriguingly, ospemifene reduced GPER1 protein and *Gper1* mRNA expression, but only during ischemia, suggesting an ambiguous role of the receptor during acute neuronal damage. For ESR2, its hypoxia/ischemia-reduced protein level was elevated after post-treatment with ospemifene, which suggests ESR2-mediated neuroprotection; however, *Esr2* silencing suggested ESR2-mediated inhibition of hypoxia-induced caspase-3 activity but not LDH release.

In conclusion, in this study, it was demonstrated for the first time that posttreatment with ospemifene has a robust neuroprotective effect against hypoxic and ischemic damage. Ospemifene-evoked neuroprotection is mediated through ESR1 (hypoxia, ischemia) and GPER1 (hypoxia) and relies on inhibiting apoptosis, as evidenced by a decrease in caspase-3 activity, a rise in mitochondrial membrane potential and normalization of apoptosis-specific factors, in addition to lowering LDH release, neurodegeneration grade, and cellular metabolic activity.

## Supplementary Information

Below is the link to the electronic supplementary material.

Supplementary file1 (DOCX 44 kb)

## Data Availability

The data that support the findings of this study are available from the corresponding author upon reasonable request. Some data may not be made available because of privacy or ethical restrictions.

## Abbreviations

ERs: Estrogen receptors
rtPA: Recombinant tissue plasminogen activator
SERMs: Selective estrogen receptor modulators
