# Supplementary material for: Posttreatment with Ospemifene Attenuates Hypoxia- and Ischemia-Induced Apoptosis in Primary Neuronal Cells via Selective Modulation of Estrogen Receptors
Source: Neurotox Res. 2023 May 2;41(4):362–79. doi: 10.1007/s12640-023-00644-5 (PMC10354152; doi:10.1007/s12640-023-00644-5)
Supplement: Supplementary file 1 — Supplementary file1 (DOCX 44 kb) [file 12640_2023_644_MOESM1_ESM.docx]

Table S1

| co-treatment | Normoxia | Normoxia + 0.1 μM ospemifene | Normoxia +  1 μM ospemifene | Normoxia +  5 μM ospemifene | Normoxia + 10 μM ospemifene |
| --- | --- | --- | --- | --- | --- |
| **LDH**  [% control] | 100,00 ± 4,56 | 102,51 ± 8,34 | 98,71 ± 8,17 | 96,74 ± 3,92 | 103,17 ± 3,85 |
| **caspase-3**  [% control] | 100,00 ± 3,41 | 99,32 ± 6,51 | 105,30 ± 6,53 | 105,32 ± 3,12 | 109,92 ± 3,69 |

Co-treatment with ospemifene (0.1, 1, 5, 10 μM) did not affect LDH release and caspase-3 activity in the normoxic conditions. The results are presented as a percentage of the normoxic control. Each value represents a mean ± SEM number of 10 to 20 replicates.

Table S2

| post-treatment | Normoxia | Normoxia + 0.1 μM ospemifene | Normoxia +  1 μM ospemifene | Normoxia +  5 μM ospemifene | Normoxia + 10 μM ospemifene |
| --- | --- | --- | --- | --- | --- |
| **LDH**  [%control] | 100,00 ± 4,62 | 108,76 ± 10,88 | 103,36 ± 11,37 | 92,43 ± 6,24 | 87,81 ± 4,51 |
| **caspase-3**  [%control] | 100,00 ± 3,18 | 100,89 ± 6,94 | 94,82 ± 6,77 | 104,56 ± 5,03 | 101,97 ± 3,17 |
| **FluoroJade C**  [%control] | 100,00 ± 1,31 | - | - | 98,62 ± 1,85 | 103,72 ± 1,31 |
| **JC-10**  [%control] | 100,00 ± 2,59 | - | - | 99,22 ± 1,01 | 97,08 ± 1,13 |
| **MTT** [%control] | 100,00 ± 1,23 | - | - | 101,72 ± 1,61 | 107,35 ± 1,23 |
| **Calcein AM** [%control] | 100,00 ± 5,65 | - | - | - | 94,09 ± 4,02 |

Post-treatment with ospemifene (0.1, 1, 5, 10 μM) did not affect LDH release, caspase-3 activity, the degree of neurodegeneration (FluoroJade-C staining), mitochondrial membrane potential (JC-10) and cell metabolic activity (MTT) in the normoxic conditions. The results are presented as a percentage of the normoxic control. Each value represents a mean ± SEM number of 10 to 20 replicates.

Table S3

| post-treatment | Normoxia | Normoxia + 0.1 μM ormeloxifene | Normoxia +  1 μM ormeloxifene | Normoxia +  5 μM ormeloxifene | Normoxia + 10 μM ormeloxifene |
| --- | --- | --- | --- | --- | --- |
| **LDH**  [% control] | 100,00 ± 8,10 | 96,85 ± 8,19 | 89,39 ± 6,56 | 101,59 ± 6,52 | 100,00 ± 8,10 |
| **caspase-3**  [% control] | 100,00 ± 2,63 | 102,98 ± 2,77 | 110,57 ± 2,15 | 109,96 ± 1,90 | 100,00 ± 2,63 |

Post-treatment with ormeloxifene (0.1, 1, 5, 10 μM) did not affect LDH release and caspase-3 activity. The results are presented as a percentage of the normoxic control. Each value represents a mean ± SEM number of 10 to 20 replicates.

Table S4

**Fold change [*Actb* normalized]**

|  | Normoxia | Normoxia + 10 μM ospemifene |
| --- | --- | --- |
| Bax | 1,001298 ± 0,025124 | 1,069395 ± 0,037184 |
| Bcl2 | 1,002720 ± 0,056373 | 1,029333 ± 0,056373 |
| Fas | 1,00469 ± 0,048786 | 1,24212 ± 0,013595 |
| Fasl | 1,007136 ± 0,059089 | 1,142124 ± 0,079032 |
| Gsk3b | 1,002716 ± 0,037168 | 1,009766 ± 0,050253 |
| Esr1 | 1,002433 ± 0,034791 | 1,306200 ± 0,063177 |
| Esr2 | 1,000360 ± 0,132366 | 1,108829 ± 0,132366 |
| Gper1 | 1,005854 ± 0,055263 | 0,803633 ± 0,031451 |
| Cyp19a1 | 1,027303 ± 0,111710 | 1,135122 ± 0,115565 |

The effects of post-treatment with ospemifene (10 μM) on expression of apoptosis- and estrogen receptor signaling-related mRNAs in the normoxic conditions. The results are presented as a percentage of the normoxic control. Each value represents a mean ± SEM of 4 to 5 replicates per group.

Table S5

**Protein level [% of the control]**

|  | Normoxia | Normoxia + 10 μM ospemifene |
| --- | --- | --- |
| BAX | 100 ± 5,33892 | 94,2486 ± 4,64904 |
| BCL2 | 100 ± 26,21332 | 86,4358 ± 15,73275 |
| FAS | 100 ± 7,68446 | 82,0394 ± 4,17775 |
| FASL | 100 ± 8,11181 | 85,5451 ± 6,06753 |
| GSK3B | 100 ± 11,38771 | 116,6665 ± 10,32634 |
| ESR1 | 100 ± 7,72808 | 144,2492 ± 21,49901 |
| ESR2 | 100 ± 15,68671 | 67,0384 ± 6,48420 * |
| GPER1 | 100 ± 8,86454 | 62,4306 ± 5,62783 |

The effects of post-treatment with ospemifene (10 μM) on expression of apoptosis- and estrogen receptor signaling-related proteins levels in the normoxic conditions. The results are presented as a percentage of the normoxic control. Each value represents a mean ± SEM of 4 to 5 replicates per group. ** p  < 0.01 compared to the normoxic cultures

**Figure S1**

Post-treatment with ormeloxifene (10 μM) further increased both, hypoxia- and ischemia-induced LDH releases. Ormeloxifene (1, 5 and 10 μM) was applied in the posttreatment paradigm. The results are presented as a percentage of the normoxic control. Each bar represents a mean ± SEM of 20 to 30 replicates. *** p  < 0.001 compared to the normoxic cultures; ^###^ p < 0.001 compared to the cultures exposed to hypoxia; ^^^ p < 0.001 compared to the cultures exposed to ischemia.

**Figure S2**

Post-treatment with ormeloxifene further increased hypoxia-induced caspase-3 activity. Ormeloxifene (1, 5 and 10 μM) was applied in the posttreatment paradigm. The results are presented as a percentage of the normoxic control. Each bar represents a mean ± SEM of 20 to 30 replicates. *** p  < 0.001 compared to the normoxic cultures; ^###^ p < 0.001 compared to the cultures exposed to hypoxia.
